# Characterization of Non-heading Mutation in Heading Chinese Cabbage (*Brassica rapa* L. ssp. *pekinensis*)

**DOI:** 10.3389/fpls.2019.00112

**Published:** 2019-02-12

**Authors:** Jingrui Li, Xiaomeng Zhang, Yin Lu, Dongxiao Feng, Aixia Gu, Shan Wang, Fang Wu, Xiangjie Su, Xueping Chen, Xing Li, Mengyang Liu, Shuangxi Fan, Daling Feng, Shuangxia Luo, Shuxin Xuan, Yanhua Wang, Shuxing Shen, Jianjun Zhao

**Affiliations:** ^1^Key Laboratory of Vegetable Germplasm Innovation and Utilization of Hebei, Collaborative Innovation Center of Vegetable Industry in Hebei, College of Horticulture, Hebei Agricultural University, Baoding, China; ^2^Plant Science and Technology College, Beijing University of Agriculture, Beijing, China

**Keywords:** *Brassica rapa*, mutant, genetic analysis, epidermis cell, RNA-Seq, phytohormones

## Abstract

Heading is a key agronomic trait of Chinese cabbage. A non-heading mutant with flat growth of heading leaves (*fg-1*) was isolated from an EMS-induced mutant population of the heading Chinese cabbage inbred line A03. In *fg-1* mutant plants, the heading leaves are flat similar to rosette leaves. The epidermal cells on the adaxial surface of these leaves are significantly smaller, while those on the abaxial surface are much larger than in A03 plants. The segregation of the heading phenotype in the F_2_ and BC_1_ population suggests that the mutant trait is controlled by a pair of recessive alleles. Phytohormone analysis at the early heading stage showed significant decreases in IAA, ABA, JA and SA, with increases in methyl IAA and *trans*-Zeatin levels, suggesting they may coordinate leaf adaxial-abaxial polarity, development and morphology in *fg-1*. RNA-sequencing analysis at the early heading stage showed a decrease in expression levels of several auxin transport (*BrAUX1, BrLAX*s, and *BrPIN*s) and responsive genes. Transcript levels of important ABA responsive genes, including *BrABF3*, were up-regulated in mid-leaf sections suggesting that both auxin and ABA signaling pathways play important roles in regulating leaf heading. In addition, a significant reduction in *BrIAMT1* transcripts in *fg-1* might contribute to leaf epinastic growth. The expression profiles of 19 genes with known roles in leaf polarity were significantly different in *fg-1* leaves compared to wild type, suggesting that these genes might also regulate leaf heading in Chinese cabbage. In conclusion, leaf heading in Chinese cabbage is controlled through a complex network of hormone signaling and abaxial-adaxial patterning pathways. These findings increase our understanding of the molecular basis of head formation in Chinese cabbage.

## Introduction

Chinese cabbage (*Brassica rapa* ssp. *pekinensis*) is an important vegetable crop of the *Brassica* genus containing several species that are of agricultural and horticultural importance. Breeding has transformed the head morphology of this crop from a loose heading to semi-heading and finally a heading type. As the edible organ, the head of Chinese cabbage is the basis for its economic value. Curling, crinkling and folding of leaves are typical characteristics of heading in Chinese cabbage. The timing and compactness of head formation are affected by the time and degree of inward curling of the leaves. Leaf polarity and phytohormones (especially auxin) are critical for leaf architecture (Liu et al., 2011), but the exact mechanism of leaf folding in Chinese cabbage is still unclear.

Leaf polarity is composed of centro-lateral axis, proximal-distal axis and abaxial-adaxial polarity (Kim and Cho, 2006). The imbalance of abaxial-adaxial polarity is important for head formation (Mao et al., 2014). Many genes involved in abaxial-adaxial polarity have been cloned in Arabidopsis, providing useful insight for exploring head development in Chinese cabbage. The *HD-ZIPIII* family genes *PHABULOSA* (*PHB*), *REVOLUTA* (*REV*) (Otsuga et al., 2001), *PHAVOLUTA* (*PHV*) (McConnell et al., 2001), and *HOMEOBOX8* (*HB8*), the transcription factors *ASYMMETRICLEAVES 1* (*AS1*) and *ASYMMETRICLEAVES 2* (*AS2*), and ta-siRNAs contribute to adaxial polarity. Auxin response factors (*ARF3*/*ETT, ARF4*), the *KANAD* gene family (*KAN1, KAN2, KAN3, YABBY* gene family) (Eshed et al., 2001; Kerstetter et al., 2001), and miRNA165/166 contribute to abaxial polarity (Palatnik et al., 2003; Hunter et al., 2006; Kidner and Timmermans, 2010; Townsley and Sinha, 2012). Although there is no heading in Arabidopsis, many genes related to abaxial-adaxial polarity in Arabidopsis also contribute to head formation in *B. rapa* (Liang et al., 2016). The re-sequencing data of different *B. rapa* and *B. oleracea* morphotypes were analyzed to detect signals of artificial selection that have shaped the complex heading trait by comparing genomic variation between heading and non-heading groups (Cheng et al., 2016). Many selection signals, or selective sweeps, including 15 loci that are under selection at syntenic positions in heading Chinese cabbages and cabbages, were detected in these two species. Several genes involved in the abaxial-adaxial patterning and leaf curvature were selected, such as *BrARF3.1, BrARF4.1, BrKAN2.1*, and *BrKAN2.3* in *B. rapa*, and *BoATHB15.2* (belonging to the *HD-ZIPIII*s) and *BoKAN2.2* in *B. oleracea*. However, the inheritance of head formation is complicated and the synergistic regulation of key genes in head development is still unclear.

Studies have shown that the synthesis, transport and signaling of phytohormones, especially auxin, play an important role in head formation in Chinese cabbage. He et al. (2000) reported that auxin participates in regulating head formation. Combined with the genome analysis of the convergence of *B. rapa* and *B. oleracea* (Cheng et al., 2016), gene enrichment analysis identified gibberellic acid (GA) biosynthesis and auxin-, cytokinin (CK)- and jasmonic acid (JA)-mediated signaling pathways. These pathways are known to be involved in leaf initiation and morphogenesis. Gao et al. (2017) found that the polar transport and uneven distribution of auxin affects head formation in Chinese cabbage. The auxin transport genes *BrLAX* (*LIKE AUXIN RESISTANT*), *BrPIN* (*PIN-FORMED*) and *BrPGP* (*P-GLYCOPROTEIN*) may also regulate the head development (Gao et al., 2017). In our previous study, the candidate genes *BrGH3.12* and *BrABF1* were identified using a Chinese cabbage-cabbage monosomic alien addition line AC_4_ by RNA-seq analysis (Gu et al., 2017). Although these phytohormone-related genes have been associated with head formation, how they communicate together to regulate this process is largely unknown. In Arabidopsis, methyl IAA ester (MeIAA) contributes to leaf curvature (Pérez-Pérez et al., 2010), while there are limited reports about how MeIAA affects the head morphology in *B. rapa*.

The reference genome of Chinese cabbage was successfully completed in 2011 (Wang et al., 2011). As a result, it revealed a whole genome triplication (WGT) event since diverging from Arabidopsis that likely facilitated the generation of extensive diversity in morphotypes (Wang et al., 2011; Cheng et al., 2014, 2016). The completion of this work has greatly promoted the study of related traits in Chinese cabbage and laid the foundation for accelerating the molecular breeding of Chinese cabbage vegetables. The genome has greatly improved our abilities to characterize mutants for gene discovery and functional research. Ethyl methanesulfonate (EMS) is the most widely used reagent for mutagenesis that provides a high mutation frequency and relatively few chromosomal aberrations. EMS mutagenesis can be used to improve specific traits and is widely used in crop germplasm resource innovation. EMS has proven to be very successful in uncovering key regulatory genes contributing to a wide range of traits in Arabidopsis (Gabrielson et al., 2006; Chiu et al., 2007). In *B. napus* and other *Brassica* crops, mutant libraries in various cultivars have been constructed by EMS mutagenesis in order to study a range of variant trait-related genes (Stephenson et al., 2010; Wang N. et al., 2010). However, in Chinese cabbage, EMS mutants are rarely used as a genetic analysis for candidate genes.

A mutant library containing 4253 M_1_ lines and the resulting M_2_ population was constructed by artificial EMS mutagenesis of the Chinese cabbage inbred line A03 (Lu et al., 2016). One flat growth non-heading mutant, *fg-1*, was obtained from the EMS-induced mutagenesis population that has flat leaves prior to the heading stage with a wrinkled leaf surface compared to the wild type. Using the mutant *fg-1* and its wild type A03, we revealed the genetic structure of the mutant heading trait in Chinese cabbage by creating segregating populations. Combining the RNA-seq and phytohormone quantifications, the molecular regulatory mechanism of head development was investigated by assessing transcript level changes and characterizing leaf growth, phytohormone levels and leaf epidermal cell morphology. In addition, a possible regulatory model is proposed. The purpose of this study was to identify new genes regulating head development in Chinese cabbage and generating new genetic resources for future Chinese cabbage crop improvement studies.

## Materials and Methods

### Plant Materials

A mutant library of Chinese cabbage was developed by EMS treatment of seeds from the inbred line A03 (Lu et al., 2016), from which a non-heading mutant of the M_5_ generation with flat growth of heading leaves (*fg-1*) was isolated. Wild type A03 has an outward-curling heading pattern on the top. The mutant *fg-1* has flat leaves during growth before the heading stage and trends to heading at the heading stage. The populations of F_1_, F_2_ and two BC_1_ (F_1_ ×*fg-1* and F_1_ × wild type) were developed from the cross between A03 and *fg-1*, which were used as the experimental materials for genetic analysis of the mutant trait. The plants were grown in a plastic tunnel on the experimental farm at Hebei Agricultural University in Baoding (115.47 E, 38.87 N), China, in 2016 and 2017.

In August 2017, 60 plants each of A03 and *fg-1* of the M_6_ generation were grown in the same plastic tunnel at Hebei Agricultural University. At the early heading stage (80 days after sowing), the 16^th^ leaf from the exterior of the developing head was sampled at four positions: apical, middle, bottom of the soft leaf and basal of the whole leaf, which were named a, b, c and d, respectively (Supplementary Figure S1). Three biological replicates were used for further analysis. All leaf samples were flash frozen in liquid nitrogen and stored at -80°C for RNA or phytohormone analysis.

### Inheritance of the Mutant Trait

The mutant *fg-1*, wild type A03, five F_1_ lines, 163 F_2_ lines, 14 F_1_ ×*fg-1* BC_1_ lines and 10 F_1_ × wild type BC_1_ lines were planted in a plastic tunnel in July 2016. To confirm the results, 145 F_2_ lines and their parents were planted again in a plastic tunnel in July 2017. The number of plants with flat growth and normal heading were counted and a Chi square test was performed. The height and expansion of plants were investigated for A03 and *fg-1*.

### Morphological Characteristics of *fg-1* at Different Developmental Stages

During the late rosette (50 days after sowing), early heading (80 days after sowing) and heading stages (90 days after sowing), the angle was measured in different layers. During the late rosette and heading stages, the leaf length, width and area of the 13^th^ and 19^th^ leaf from the exterior was measured via ImageJ. At the heading stage, the expansion degree (the maximum distance of the outside leaves) and the plant height were measured for *fg-1* and the wild type.

### Analysis of Leaf Abaxial and Adaxial Epidermal Cell Structure and Area

During the rosette (40 days after sowing) and heading stages, 2 mm × 2 mm from the top and the middle edge of leaves were prepared for abaxial epidermal cell structure and area imaging by scanning electron microscopy. Leaves were fixed in 2.5% amyl glycol at room temperature for 24 h then rinsed completely with 0.1 M phosphate buffer and fixed with 1% osmium acid for 2 h. The samples were dehydrated with an alcohol gradient (30, 50, 70, 80, 90, 95, and 100%) followed by an overnight treatment with isoamyl acetate. Samples were dried using a LEICA EM CPD 030 critical point drying. An Eiko IB5 Ion Coater instrument was used to spray gold after the sample was glued to the table. A Hitachi SU-8010 scanning electron microscope was used to observe and photograph the samples.

During the late heading stage (100 days after sowing), the adaxial epidermis cell structure at the top and the middle edge of the leaves were observed using the glue-marking method. The glue was evenly coated on the leaves. After air-drying, the cells were removed by tweezers and spread on the slide for imaging. Cell structure was examined using the optical microscope (Olympus CX41, Tokyo, Japan). The area of the cells was measured using ImageJ software (Collins, 2007).

### RNA Extraction, cDNA Library Preparation and Sequencing for RNA-Seq

Total RNA was isolated using an RNA extraction kit (TIANGEN, China). RNA purity and concentration were assessed as previously described (Zhao et al., 2014). The cDNA library was prepared and sequenced as previously described (Zhao et al., 2014; Gu et al., 2017).

### RNA-Seq Data Analysis

Raw reads were filtered by removing adaptors, poly(N) and low quality reads (Li et al., 2015). The reference genome and gene model annotation files were downloaded from the *B. rapa* database (^1^v1.5). Filtered reads were mapped to the genome using HISAT2 v2.0.5 (Kim et al., 2015). HTSeq v0.6.0 (Anders, 2010) was used to generate counts per gene. The FPKM values of each gene were calculated based on the gene length and read counts. Differential expression analysis of each set of two groups (three biological replicates per group) was performed using the DESeq R package (1.16.1.) (Anders and Huber, 2010, 2012; Wang L. et al., 2010). The *P*-values were adjusted using the Benjamini and Hochberg (1995). method Genes with a corrected *P* < 0.05 were considered differentially expressed.

### Validation of RNA-Seq by qRT-PCR

Quantitative real-time PCR (qRT-PCR) was carried out as previously described (Zhao et al., 2014; Gu et al., 2017; Zhang et al., 2017). To verify the RNA-seq data, we analyzed the expression of ten genes by qRT-PCR. A Chinese cabbage *actin* gene (Bra009081) was used as an internal reference. The 2^-[Ct-Ct(actin)]^ × 1000 method was used to calculate gene relative expression levels between wild type and *fg-1*. Primers are listed in Supplementary Table S1. Each experiment was repeated three times.

### Concentration Analysis of Different Phytohormones in *fg-1* and Wild Type

The b sections of (Supplementary Figure S1) the 16^th^ leaf from the exterior at the early heading stage were harvested, weighed and immediately frozen in liquid nitrogen. Samples (120 mg) were ground into a fine powder and extracted with methanol/water (v/v = 8/2) at 4°C. The concentrations of six classes of phytohormones [auxin, abscisic acid (ABA), GA, salicylic acid (SA), JA and CK], including 25 forms were analyzed using a high-pressure liquid chromatography-electrospray tandem mass spectrometry (LC-ESI-MS/MS) system as previously described (Wang et al., 2018). Three technical replicates and three biological replicates were conducted.

## Results

### Inheritance of Mutant Trait

Genetic analysis revealed that the phenotypic traits of all F_1_ plants were similar to the wild type. In the F_2_ populations of years 2016 and 2017, the proportions of wild type and mutant plants were 3.17:1 and 3.68:1, respectively, conforming to the 3:1 ratio (Chi square test: χ^2^ = 0.691 and 0.311). In the backcross population F_1_ × *fg-1*, the proportion of wild type and mutant plants was 1.33:1, conforming to the 1:1 ratio (Chi square test: χ^2^ = 0.593), and all plants in the backcross group F_1_ × wild type were similar to wild type (Table 1). This phenotypic analysis suggests that the flat growth trait is controlled by a pair of recessive alleles.

**Table 1:** Segregation ratios of F_1_, F_2_ and two BC_1_F_1_s between *fg-1* and wild type.

Generation	Total plants	Wild type plants (heading)	Mutant plants (non-heading)	Segregation ratio	χ^2^ value
F_1_	5	5	0	5:0	
F_2_ (2016)	163	124	39	3.17:1	0.691
F_2_ (2017)	145	114	31	3.68:1	0.311
F_1_ ×*fg-1*	14	8	6	1.33:1	0.593
F_1_ × wild type	10	10	0	10:0	

### Morphological Characteristics of *fg-1* During Leaf Development

**FIGURE 1:**
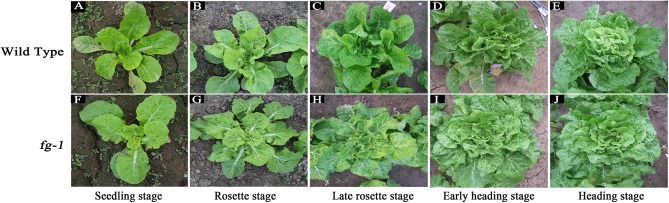
Morphological characteristics of the wild type and *fg-1* at different developmental stages. **(A–E)** represent *fg-1* plants at seedling, rosette, late rosette, early heading and heading stages, respectively. **(F–J)** represent wild type plants at seedling, rosette, late rosette, early heading, and heading stages, respectively.

During whole leaf development, all *fg-1* expanded leaves show flat growth with a reduced leaf angle to the ground (Supplementary Table S2). All *fg-1* leaves are visibly wrinkled and lacking the outward-curling features seen in wild type. In the wild type, the angles from rosette leaves to the ground are larger (Supplementary Table S2). Additionally, the rosette leaves are slightly wrinkled; the heading leaves are outward-curling at the top edge. From the early heading stage, the newly expanded leaves curl transversely and longitudinally to form the head (Figure 1).

During the late rosette and heading stages, the leaf length and area in *fg-1* are significantly smaller than that in wild type, while there is no significant difference in leaf width (Supplementary Table S3). The leaf shape in *fg-1* tends to be obovate, but that in wild type is close to rectangle.

At the heading stage, the expansion degree of the mutant plants (the maximum distance between the outside leaves) was similar to that of the wild type, while plant height was significantly less (Supplementary Figure S2).

### Leaf Abaxial and Adaxial Epidermal Cell Structure and Area in *fg-1*

**FIGURE 2:**
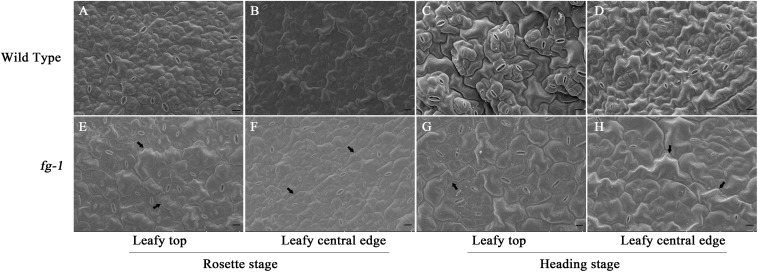
Scanning electron microscopy for abaxial epidermis cells of leaf sections at different developmental stages for the wild type and *fg-1*. **(A,B)** represent abaxial epidermis cells of the top and central leaf edge at the rosette stage in wild type. **(C,D)** represent abaxial epidermis cells of the top and central leaf edge at the heading stage in wild type. **(E,F)** represent abaxial epidermis cells of the top and central leaf edge at the rosette stage in *fg-1*. **(G,H)** represent abaxial epidermis cells of the top and central leaf edge at the heading stage in *fg-1.* Slender cells are indicated by arrows. Length of black bars is 10 μm.

To further characterize the difference between the leaf morphology of wild type and *fg-1*, abaxial epidermal cells of leaf sections were observed by scanning electron microscopy at the rosette and heading stage (Figure 2). Adaxial epidermal cells of leaf sections were also observed at the late heading stage (Supplementary Figure S3). At both stages, more large and slender cells in the abaxial epidermis were observed at the top and central edge of *fg-1* mutant leaves compared to wild type. The abaxial epidermis cell areas of the top and the central leaf edges in *fg-1* were significantly greater than that in the wild type at the rosette and heading stages (Table 2). At the rosette stage, abaxial epidermis cell area of both the top and the central edge of the leaf in *fg-1* was 57.7 and 28.9% larger than that in the wild type, respectively. At the heading stage, the abaxial epidermis cell area of both the top and central edge was 35.8 and 39.1% larger than that of the wild type, respectively (Table 2). At the late heading stage, the adaxial epidermis cell area of both the top and central edge was 26.1 and 41.1% lower than that of the wild type, respectively (Table 3).

**Table 2:** Area of leaf abaxial epidermal cells.

Type	Rosette stage (μm^2^)		Heading stage (μm^2^)	
	Leafy top	Leafy central edge	Leafy top	Leafy central edge
Wild type	380.2 ± 40.3 b	423.0 ± 41.7 b	735.6 ± 114.8 b	768.7 ± 67.8 b
*fg-1*	899.3 ± 118.5 a	595.1 ± 50.4 a	1145.2 ± 109.0 a	1261.5 ± 125.1 a

*Each value is the mean ± SE for 60 cells from three independent replicates. Different letters indicate significant differences between treatments (*P* < 0.05) according to Duncan’s multiple range test*.

**Table 3:** Area of leafy adaxial epidermal cells in the late heading stage.

Type	Leafy top (μm^2^)	Leafy central edge (μm^2^)
Wild type	3058.4 ± 237.8 a	3520.9 ± 352.3 a
*fg-1*	2425.7 ± 141.2 b	2496.2 ± 266.4 b

*Each value is the mean ± SE for 60 cells from three biological replicates. Different letters indicate significant differences between treatments (*P* < 0.05) according to Duncan’s multiple range test*.

### Identification of Differentially Expressed Genes via RNA-seq Between *fg-1* and A03 Leaves

Following read processing and quality filtering, roughly 6.34 Gb remained for each sample with over 85% of reads uniquely mapped (Supplementary Table S4). We identified 7669 DEGs in the top leaf section, with 3966 down-regulated and 3703 up-regulated; 5022 DEGs were found in the middle section, with 2625 down-regulated and 2397 up-regulated; 3907 DEGs were found in the base of the soft leaf section, with 1900 down-regulated and 2007 up-regulated; and 4823 DEGs were found in the basal section, with 2090 down-regulated and 2733 up-regulated (Supplementary Table S5). To validate the RNA-seq data, 10 representative DEGs were assessed by qRT-PCR analysis. The results indicated that the expression of the genes between the two methods were consistent (*R*^2^ = 0.8441) (Supplementary Figure S4).

### Pathway Analysis Involved in Head Formation in Chinese Cabbage

**FIGURE 3:**
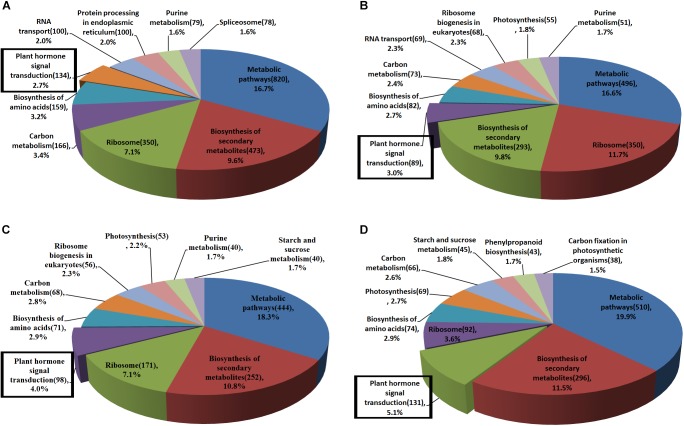
The main KEGG pathways of DEGs in *fg-1* compared with wild type. Different colors represent the pathway names. The number of genes involved in each pathway is labeled in parentheses. Pathways in the rectangular boxes are involved in plant hormone signal transduction. **(A–D)** represent the main KEGG pathways of DEGs in sections a–d of the *fg-1* leaf compared with the wild type, respectively.

**FIGURE 4:**
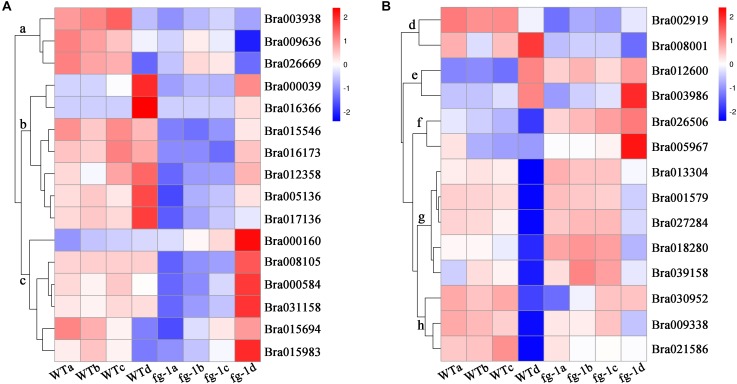
Expression profile of the auxin influx and efflux carrier genes, *IAMT1* and *TCP*s. The bar on the right side of the heat map represents relative expression values where 2, 0, and –2 represent high, intermediate and low expression, respectively. Red indicates relatively high gene expression levels and deep blue indicates relatively low gene expression levels. **(A)** The expression differences in auxin influx and efflux carrier genes in four leaf sections of the *fg-1* mutant and wild type. **(B)** The expression differences in *BrIAMT1* and *BrTCP* genes in four leaf sections of the *fg-1* mutant and wild type.

Pathway definitions were derived from the KEGG (Kyoto Encyclopedia of Genes and Genomes) database (Figure 3). Among the main pathways of DEGs, plant hormone signal transduction has a known role in regulating leaf development. Except for genes *BrLAX2, BrPIN1*, and *BrPIN6* in leaf section d (Figure 4A, subcluster c), the expression levels of the auxin influx and efflux carrier genes, three *BrAUX1* genes, four *BrLAX*s and six *BrPINs* were reduced (Figure 4A and Supplementary Tables S5, S6). Additionally, the main expression profiles of genes (including *BrARF7*, 19 *BrAUX*/*IAA*s, six *BrGH3* and 50 *BrSAUR*s) involved in IAA signaling were also down-regulated (Supplementary Figure S6A, subcluster b and c; Supplementary Table S7), but the expression levels of three syntenic genes of *AtARF5* in leaf section d were elevated in *fg-1* (Supplementary Figure S6A, subcluster a; Supplementary Table S7). The expression patterns of 30 genes (including 10 *BrPYR*/*PYL*s, nine *BrPP2C*s, six *BrSnRK2*s and five *BrABF*s) in abscisic acid (ABA) signaling (Supplementary Figure S6B, subcluster b; Supplementary Table S7), 19 genes (including three *BrHK3* genes, three *AHP*s, two *BrB-ARR*s and 11 *BrA-ARRs*) in CK signaling (Supplementary Figure S7A), 16 genes (including three *BrJAR1* syntenic genes, two *BrCOI1* syntenic genes, 10 *BrJAZ*s and one *BrMYC2*) in JA signal transduction (Supplementary Figure S7B) and 12 genes (nine *BrTGA*s and three *BrNRR*s) in SA signal transduction (Supplementary Figure S7C) were altered.

However, the expression levels of *BrIAMT1* (Bra002919) encoding the IAA carboxyl methyltransferase 1, known to alter leaf curvature phenotypes through an auxin-regulated developmental process (Qin et al., 2005), decreased in three sections (a, b, c) of *fg-1* leaves (Figure 4B, subcluster d; Supplementary Figure S5). In addition, except for three *BrT* in subcluster h and *BrTCP22* (Bra008001) in subcluster d (Figure 4B and Supplementary Figure S5), increased expression levels of the remaining nine *BrTCP* genes that are known to regulate leaf curvature were observed in the *fg-1* a, b and c leaf sections (Figure 4B and Supplementary Table S6). Additionally, 19 genes regulating leaf abaxial-adaxial patterning, including *BrKAN1, BrBOP2* (*BrNPR5*), six *BrHD-ZIPIII*s (*BrREV, BrHB8.1, BrHB8.2, BrHB9, BrHB14.1, BrHB14.2*), four *BrYAB*s (*BrYAB1.1, BrYAB1.2, BrYAB2, BrYAB3*) and seven *BrKNOX* class II genes (*BrKNAT1, BrKNAT2, BrKNAT3, BrKNAT4.1, BrKNAT4.2, BrKNAT5, BrKNAT6*), were differentially expressed in several sections of *fg-1* leaves (Supplementary Figure S8 and Supplementary Table S9).

### Phytohormone Quantification in *fg-1* and Wild Type

**FIGURE 5:**
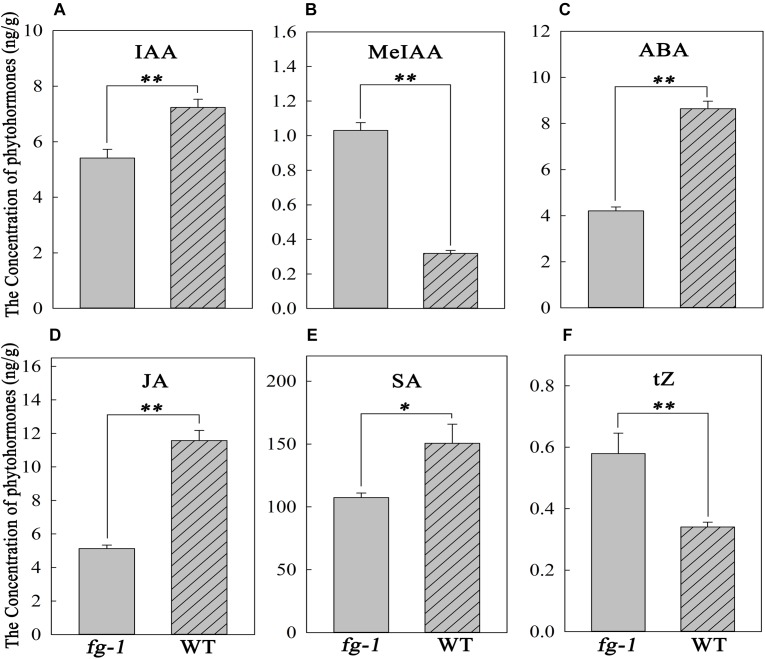
Concentrations of IAA, MeIAA, ABA, JA, SA and tZ in the *fg-1* and wild type. An asterisk represents a significant difference (^∗^*p* < 0.05 and ^∗∗^*p* < 0.01). **(A–F)** show the concentrations of IAA, MeJA, ABA, JA, SA and tZ in *fg-1* and wild type, respectively.

Concentrations of auxin, including IAA and MeIAA, ABA, salicylic acid (SA), JA and CK, including *trans*-Zeatin (tZ), in *fg-1* were significantly different from wild type. Specifically, in *fg-1*, levels of IAA, ABA, JA and SA were reduced 25.2, 51.3, 55.7, and 28.7% compared with A03, respectively (Figure 5A,C–E). However, levels of MeIAA and tZ were increased in *fg-1* to 3.2 and 1.7 fold that of the wild type, respectively (Figure 5B,F). The levels of other phytohormones such as GA showed no difference between *fg-1* and wild type.

## Discussion

**FIGURE 6:**
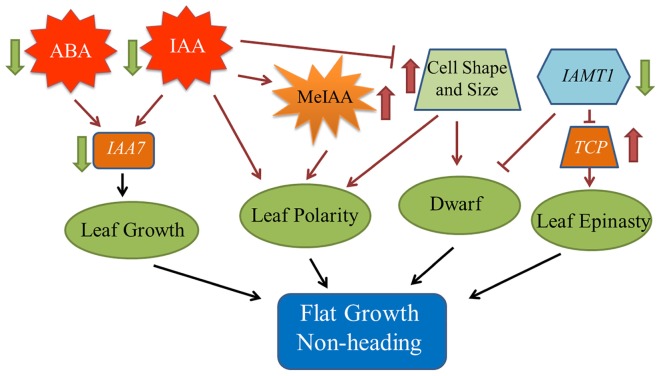
Model of the regulatory network contributing to the *fg-1* non-heading leaf phenotype. The black solid arrows denote the findings inferred from the present study. Dark red arrows denote the results of previous studies.

To explore the heading mechanism in Chinese cabbage, a comprehensive regulatory network was constructed based on the above results (Figure 6).

### Epidermal Cell Shape Variation Between *fg-1* and Wild Type

Leaf shape, such as curvature and wrinkling, is closely related to cell structure (Liu et al., 2011). Leaves also have unique abaxial-adaxial characteristics, including the distribution of hair and stoma on the surface of the leaf and the type and arrangement of the cells on the abaxial side of the leaf (Tsukaya, 2002). The Arabidopsis *hyl1* mutant lacks the long and narrow cells in the abaxial epidermis, resulting in dramatically inward curling leaves (Liu et al., 2011). Interestingly, similar to the inward curling phenotype in Arabidopsis *hyl1*, leaves of wild-type Chinese cabbage also curl inward and form a head due to the lack of these long and narrow cell types in the abaxial epidermis. The presence of elongated and narrow cells in the abaxial epidermis leads to the non-heading flat growth of *fg-1* leaves (Figure 2 and Table 1). The single adaxial cell area in wild type is significantly larger than in *fg-1* (Table 3 and Supplementary Figure S3), suggesting that cell shape and size play an important role in maintaining leaf polarity and regulating heading in Chinese cabbage (Figure 6).

### The Role of Phytohormones in Leaf Development

Phytohormones play essential roles in regulating leaf shape and polarity (Gazzarrini and Mccourt, 2003; Santner and Estelle, 2009; Stamm and Kumar, 2010; Cheng et al., 2016). Four phytohormones in particular (CK, auxin, GA and JA) have been implicated in heading morphotypes of both Chinese cabbage and cabbage (Cheng et al., 2016). Auxin directly influences cell growth (Teale et al., 2006) but also contributes to axial polarity and patterning of leaves (Barkoulas et al., 2007). IAA is the predominant form of auxin in plants (Teale et al., 2006), while MeIAA, a non-polar and more potent auxin based on hypocotyl elongation assays, plays an important role in regulating auxin homeostasis and plant development (Qin et al., 2005). In *fg-1*, IAA homeostasis was altered compared to wild type with lower levels of IAA and elevated levels of MeIAA (Figure 5A,B). These differences might contribute to the disrupted axial polarity (Figure 1) and cell enlargement observed in *fg-1* leaves (Figure 2). IAA transport and signaling play essential roles in controlling plant growth and development (Teale et al., 2006). Consistent with the reduced accumulation of IAA, the expression levels of auxin influx and efflux carrier genes were reduced, similar to the majority of auxin responsive genes (Figure 4A and Supplementary Figure S6A), providing further support for the importance of auxin in head formation.

Endogenous ABA is involved in plant responses to environmental stresses in addition to fine-tuning plant development through a regulatory circuit of primary metabolism, cell growth and cell division (Fedoroff, 2002; Himmelbach et al., 2003). Cross-talk between ABA and auxin signaling occurs via *AXR2*/*IAA7* (Timpte et al., 1994; Nagpal et al., 2000; Fedoroff, 2002). The homeostasis of ABA is also disturbed in *fg-1* (Figure 5C), along with changes in ABA signaling (Supplementary Figure S6B). The expression of two *AtIAA7* syntenic genes in subclusters b and c were significantly down-regulated in *fg-1* (Supplementary Figure S6A) suggesting that the cross-talk between ABA and IAA signaling impacts leaf heading (Figure 6).

CKs have profound roles in plant growth regulation, such as release of lateral buds from apical dominance and delay of senescence (Kasahara et al., 2004). As a form of CKs, tZ is active and regulates plant development (Inoue et al., 2001; Yamada et al., 2001). In this study, tZ accumulates (Figure 5F) in *fg-1* suggesting that higher concentrations of tZ might delay leaf senescence via CK signaling resulting in the non-heading phenotype. At the late heading stage of *fg-1* plants, leaves begin heading, but under suboptimal conditions (e.g., low temperature and short day length), the leaves do not form a complete head (Figure 1).

SA has crucial roles in regulating physiological processes via a complex SA signaling network (Vanacker et al., 2001; Vicente and Plasencia, 2011). Altered SA levels disturb normal growth phenotypes (e.g., plant stature, leaf morphology, and cell size) (Greenberg et al., 2000; Song et al., 2004; Brodersen et al., 2005). JA regulates plant stress response, growth and development (e.g., leaf movement and senescence) through a regulatory network that integrates other plant hormones including SA, IAA and ABA (Nakamura et al., 2006; Wasternack, 2007; Wasternack and Strnad, 2016). In *fg-1*, the accumulation of both JA and SA was reduced, with up-regulated expression patterns of several genes involved in JA and SA signaling (Figure 5D,E and Supplementary Table S8). The abnormal leaf growth and development seen in *fg-1* could be due to disrupted coordination of multiple hormone signals.

In leaf section b of *fg-1*, the expression patterns of four *BrPYR*/*PYL*s, four *BrPP2Cs*, three *BrSnRK2*s and *BrABF3* involved in ABA signaling were up-regulated, which was contrary to the change of ABA content. This same trend of gene expression patterns was observed in JA and SA signaling pathways in the *fg-1* leaf section b. One explanation for this discrepancy is that these genes may be involved in multifunctional roles in other signaling pathways important for leaf development (Wasternack and Strnad, 2016).

### Impacts of *BrIAMT1* and *BrTCP*s on Leaf Polarity in Chinese Cabbage

The role of *IAMT1* in Arabidopsis leaf development was implicated in an auxin-regulated developmental process (Qin et al., 2005). Decreased expression levels of *IAMT1* causes dramatic epinastic leaf phenotypes (including smaller leaves and dwarfism) in *IAMT1*-RNAi Arabidopsis plants (Qin et al., 2005). Interestingly, the expression level of *BrIAMT1* (Bra002919) was also down-regulated in *fg-1* (Figure 4A, subcluster d) with a similar axial patterning of leaves and plant dwarfism as shown in *IAMT1-*RNAi Arabidopsis plants.

A subset of *TCP* genes (e.g., *TCP2, TCP3, TCP4*, and *TCP10*) have been shown to play important roles in regulating leaf morphology (Nath et al., 2003; Palatnik et al., 2003; Mao et al., 2014). Higher *IAMT1* expression levels were correlated with lower expression of the *TCP* genes in Arabidopsis *iamt1-D* mutants that display dramatic hyponastic leaf phenotypes (Qin et al., 2005). In contrast, decreased mRNA levels of *BrIAMT1* and increased expression of nine *BrTCP* genes (Figure 4B, subcluster e, f and g) may contribute to the epinastic growth of leaves in *fg-1* (Figure 6).

It has been reported that the enzyme IAMT1 converts IAA to MeIAA *in vitro* (Qin et al., 2005; Zhao et al., 2008). Nevertheless, MeIAA is hard to detect (Qin et al., 2005), and it is unclear whether plants make MeIAA or whether IAMT1 could catalyze IAA to MeIAA *in vivo* (Zimmerman and Hitchcock, 1937; Yang et al., 2008). In this study, the expression levels of *IAMT1* were decreased in leaf sections a, b and c of *fg-1*, while the concentration of MeIAA, detected via the HPLC-ESI-MS/MS system, was elevated in *fg-1* (Figure 5). There are two possible explanations for these results. First, the IAMT1 enzyme function may be distinct between *in vitro* and *in vivo* conditions. Second, the gene underlying the *fg-1* mutant may disrupt the BrIAMT1 catalysis in methylating IAA to MeIAA. The altered hormone levels and expression patterns of hormone responsive genes suggests a complex interplay of hormone signaling is necessary for proper head formation. Deciphering this complex network through additional genetic and genomic studies is needed to inform future breeding efforts for head morphology in Chinese cabbage.

## Author Contributions

JL and XZ performed the research and wrote the manuscript. DxF and XS surveyed the morphological characteristics. ML, AG, and SX analyzed the data of RNA-Seq. SL, SW, and YL performed the genetic analysis. DlF, XL, and FW performed the morphology analysis. XC and SF reviewed the manuscript. JZ, SS, and YW designed the research and reviewed the manuscript. All authors declare no competing financial interests.

## Conflict of Interest Statement

The authors declare that the research was conducted in the absence of any commercial or financial relationships that could be construed as a potential conflict of interest.

## Footnotes

1 http://brassicadb.org/brad/index.php
