# Supplementary material for: Characterization of Non-heading Mutation in Heading Chinese Cabbage (Brassica rapa L. ssp. pekinensis)
Source: Front Plant Sci. 2019 Feb 12;10:112. doi: 10.3389/fpls.2019.00112 (PMC6379458; doi:10.3389/fpls.2019.00112)
Supplement: Supplementary file 1 [file Data_Sheet_1.zip › Data Sheet 1/Supplementary figure legends.docx]

**Characterization of non-heading mutation in heading Chinese cabbage (*Brassica rapa* L. ssp. *pekinensis*)**

Jingrui Li^#, 1^, Xiaomeng Zhang^#, 1^, Yin Lu^1^, Dongxiao Feng^1^, Aixia Gu^1^, Shan Wang^1^, Fang Wu^1^, Xiangjie Su^1^, Xueping Chen^1^, Xing Li^1^, Mengyang Liu^1^, Shuangxi Fan^2^, Daling Feng^1^, Shuxin Xuan^1^, Shuangxia Luo^1^, Yanhua Wang*^,1^, Shuxing Shen*^,1^ and Jianjun Zhao*^,1^

^1^ Key Laboratory of Vegetable Germplasm Innovation and Utilization of Hebei, Collaborative Innovation Center of Vegetable Industry in Hebei, College of Horticulture, Hebei Agricultural University, Baoding, China, ^2^ Plant Science Technology College, Beijing University of Agriculture, Beijing 102206, China

^#^Authors contributed equally to this work.

* Correspondence:

Jianjun Zhao

jjz1971@aliyun.com;

Yanhua Wang

yywyh@hebau.edu.cn;

Shuxing Shen

shensx@hebau.edu.cn

**Supplementary Figure S1. Samples of Chinese cabbage leaf.** Sections a, b and c represent the apical, middle and bottom sections of soft leaves. Section d represents the basal section of the whole leaf.

**Supplementary Figure S2. Plant height and plant expansion of A03 and *fg-1*.**

**Supplementary Figure S3. Adaxial epidermis cells in different leaf sections in late heading stage for wild type and *fg-1*.** A and B represent adaxial epidermis cells in leafy top and central edge in wild type, C and D represent adaxial epidermis cells in leafy top and central edge in *fg-1*. Length of black bars is 50 μm.

**Supplementary Figure S4. Comparison between RNA-Seq and qRT-PCR data for 10 genes expressed in four different leaf sections of *fg-1* and wild type.**

**Supplementary Figure S5. The qRT-PCR results of eight important genes in the regulation of leaf heading in Chinese cabbage.** Red and blue columns represent relative expression levels of genes in wild type and *fg-1*, respectively. An asterisk represents a significant difference (*, p < 0.05; **, p < 0.01).

**Supplementary Figure S6. Clustering analysis of the expression profiles of the DEGs involved in auxin and ABA signaling.** The bar on the right side of the heat map represents relative expression values; therefore, values 2, 0 and 2 represent high, intermediate and low expression, respectively. Red indicates relatively high gene expression levels, and deep blue indicates relatively low gene expression levels.

**Supplementary Figure S7. Clustering analysis of the expression profiles of the DEGs involved in CK, JA and SA signaling.** The bar on the right side of the heat map represents relative expression values; therefore, values 2, 0 and 2 represent high, intermediate and low expression, respectively. Red indicates relatively high gene expression levels, and deep blue indicates relatively low gene expression levels.

**Supplementary Figure S8. Clustering analysis of the expression profiles of the DEGs involved in leaf polarity.** The bar on the right side of the heat map represents relative expression values; therefore, values 2, 0 and 2 represent high, intermediate and low expression, respectively. Red indicates relatively high gene expression levels, and deep blue indicates relatively low gene expression levels.
