# Supplementary figures and images for: Characterization of Non-heading Mutation in Heading Chinese Cabbage (Brassica rapa L. ssp. pekinensis)
Source: Front Plant Sci. 2019 Feb 12;10:112. doi: 10.3389/fpls.2019.00112 (PMC6379458; doi:10.3389/fpls.2019.00112)

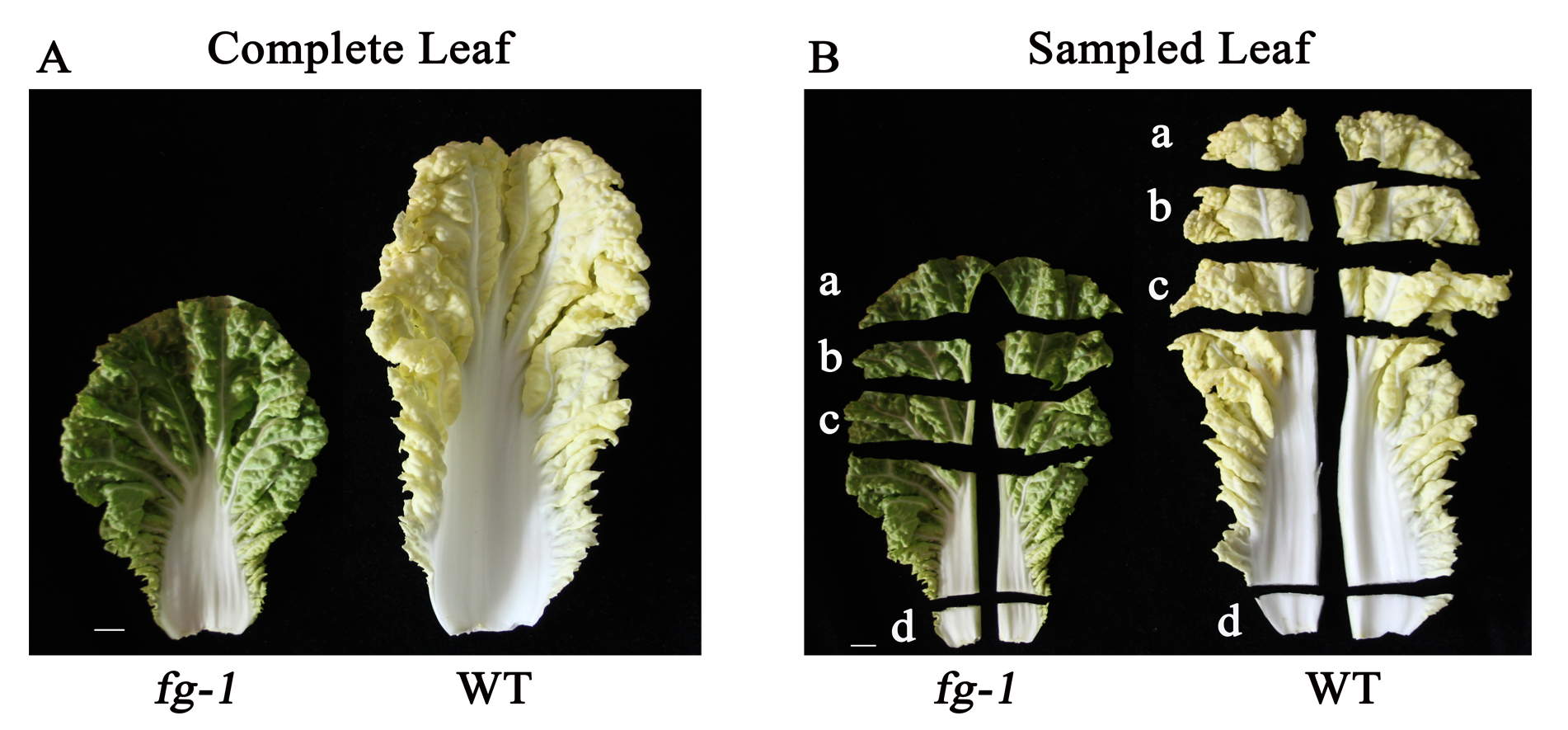

Supplement: Supplementary file 1 [file Data_Sheet_1.zip › Data Sheet 1/Supplementary Figure 1.tif]

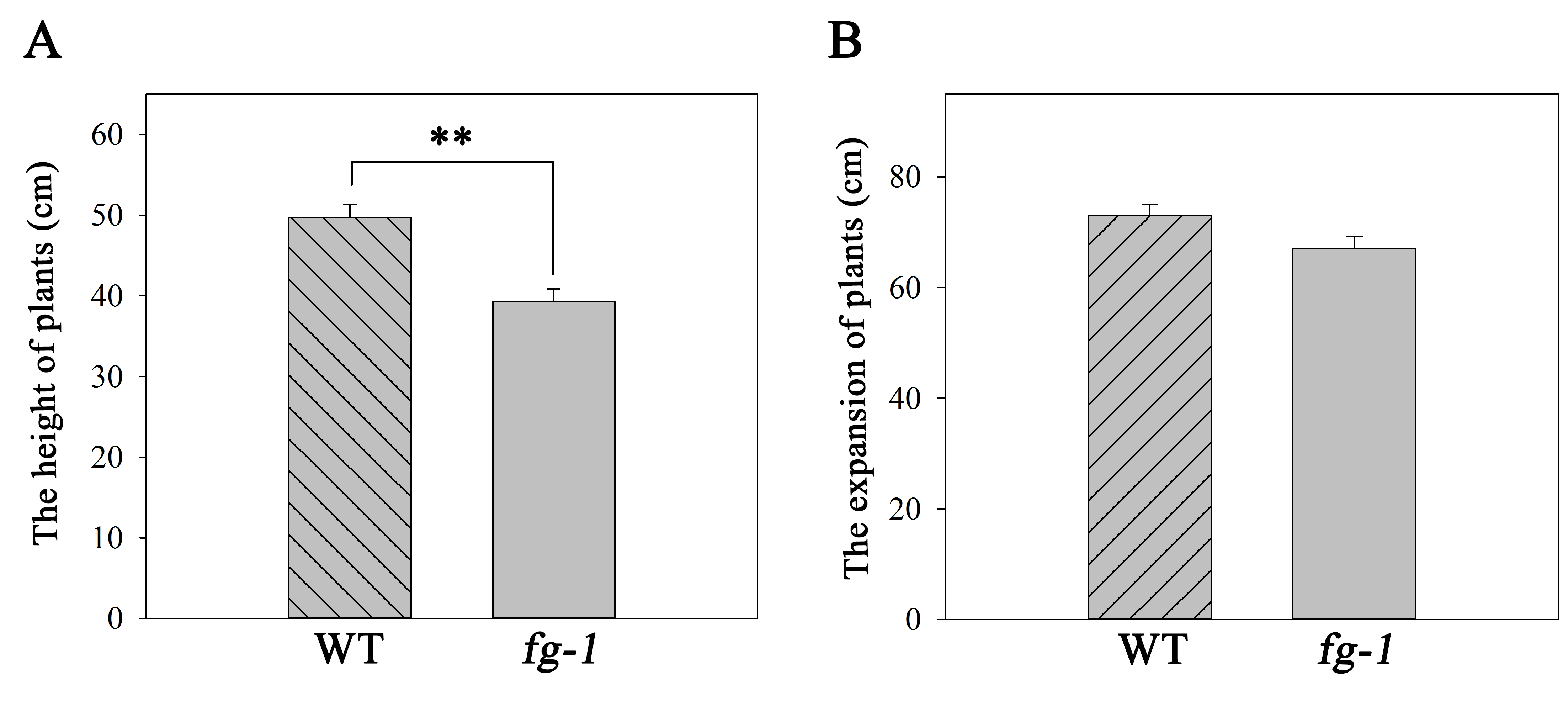

Supplement: Supplementary file 1 [file Data_Sheet_1.zip › Data Sheet 1/Supplementary Figure 2.tif]

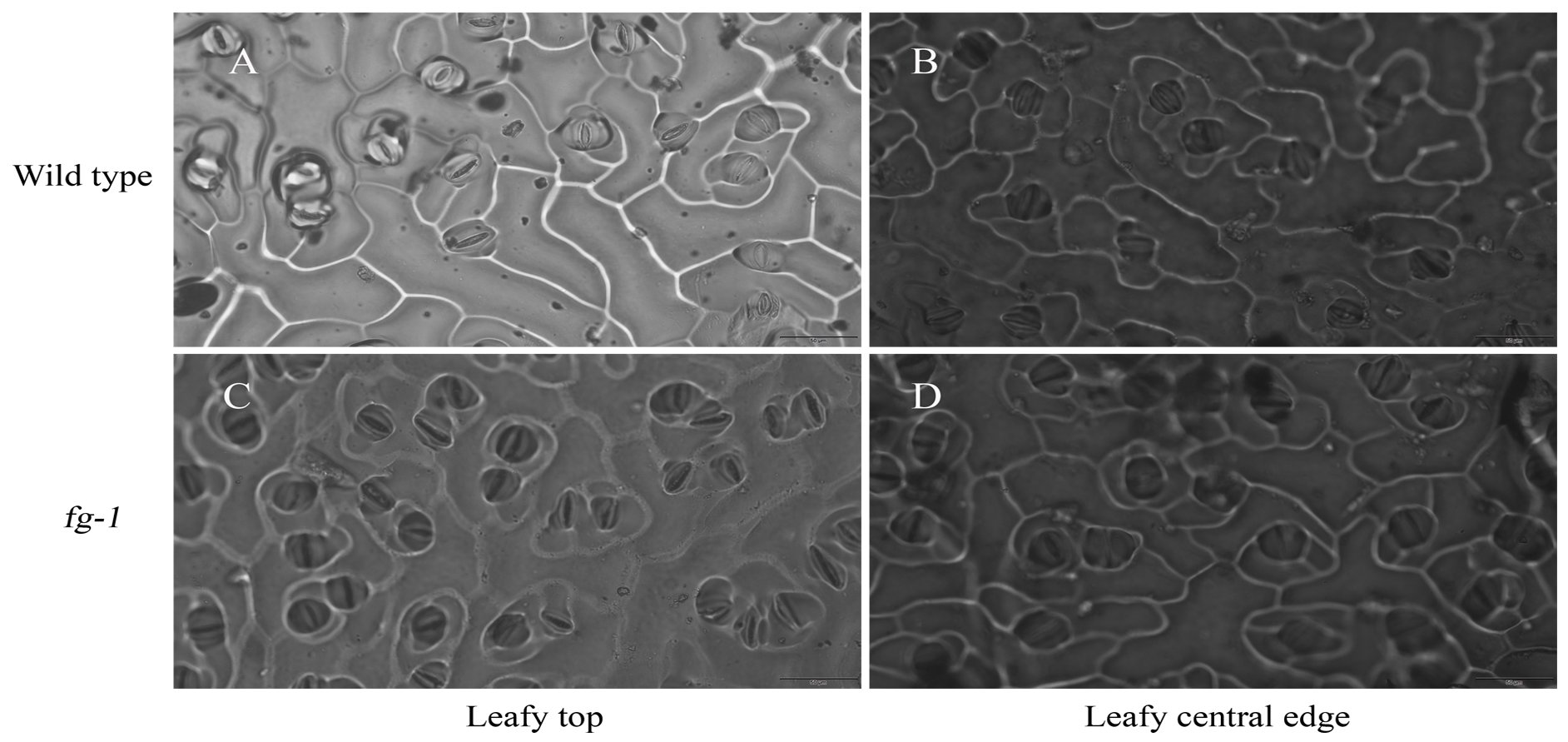

Supplement: Supplementary file 1 [file Data_Sheet_1.zip › Data Sheet 1/Supplementary Figure 3.tif]

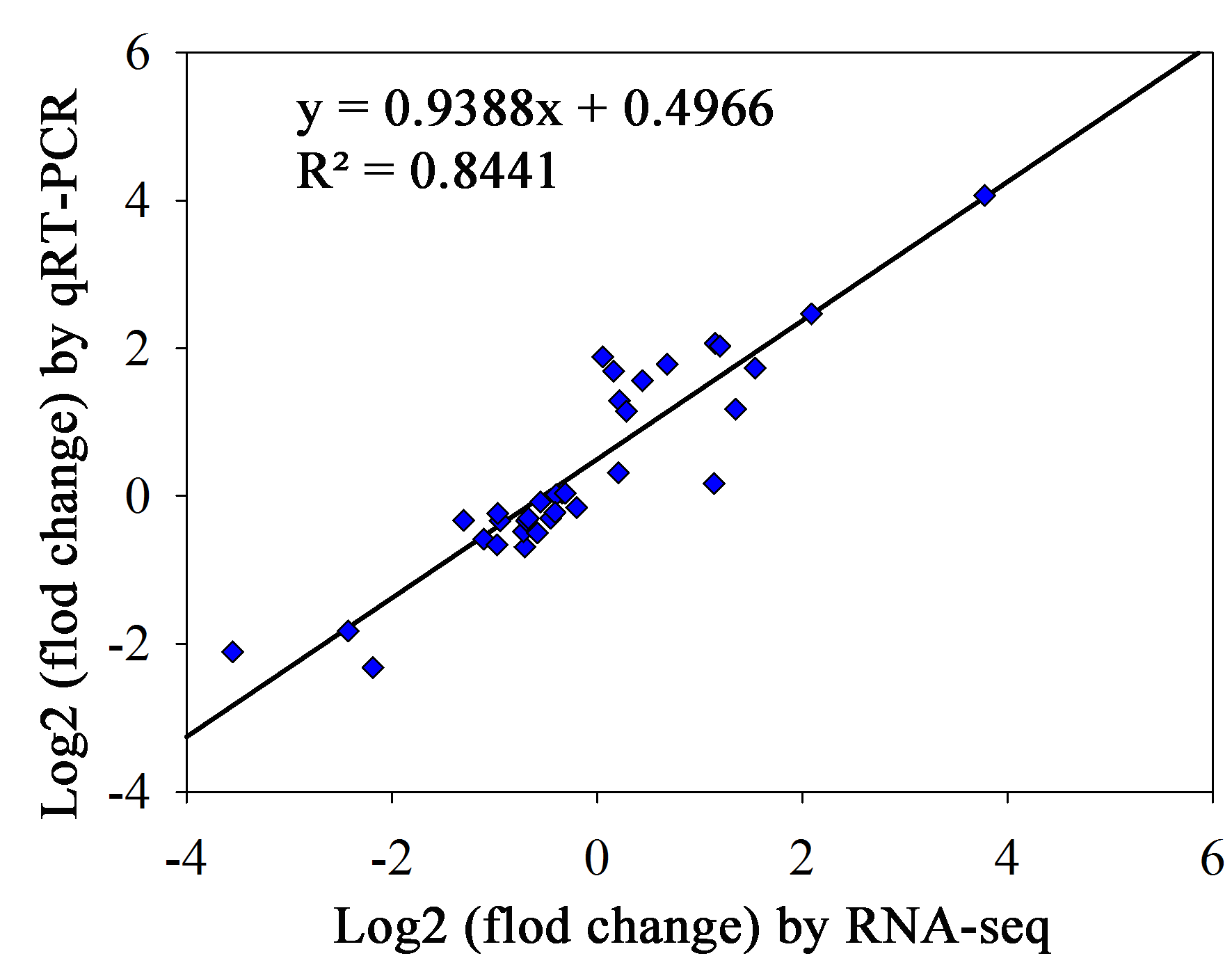

Supplement: Supplementary file 1 [file Data_Sheet_1.zip › Data Sheet 1/Supplementary Figure 4.TIF]

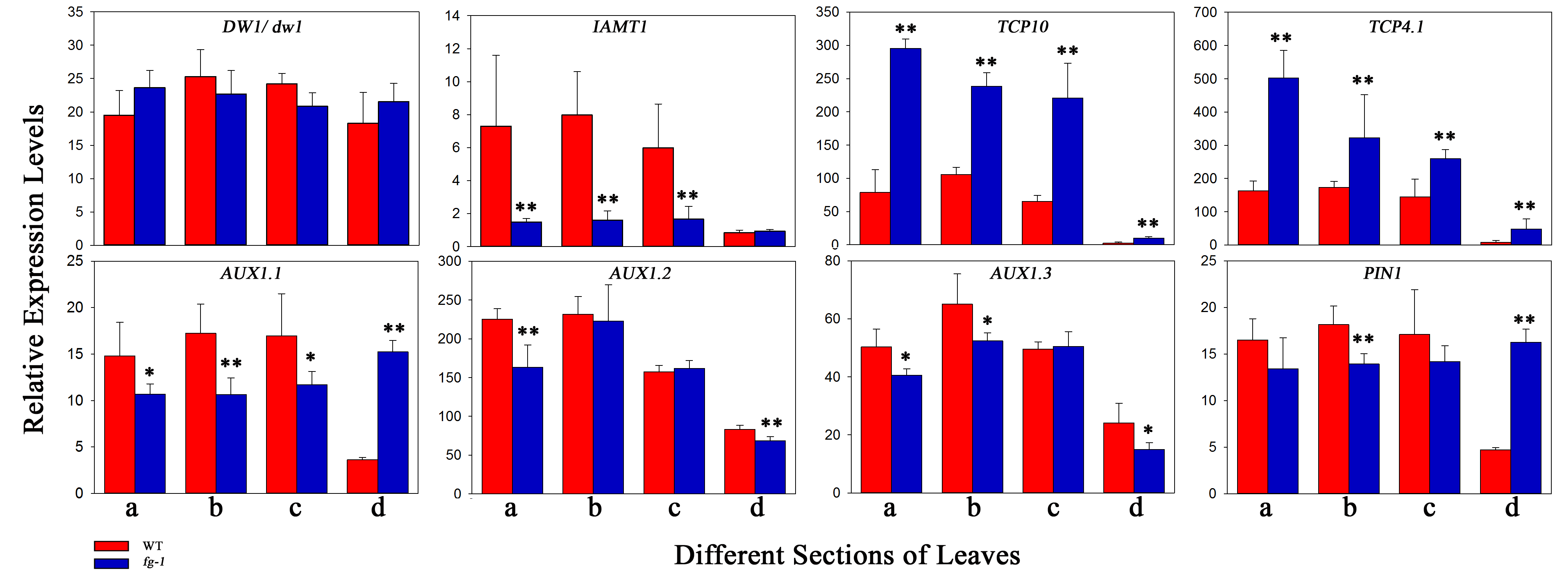

Supplement: Supplementary file 1 [file Data_Sheet_1.zip › Data Sheet 1/Supplementary Figure 5.tif]

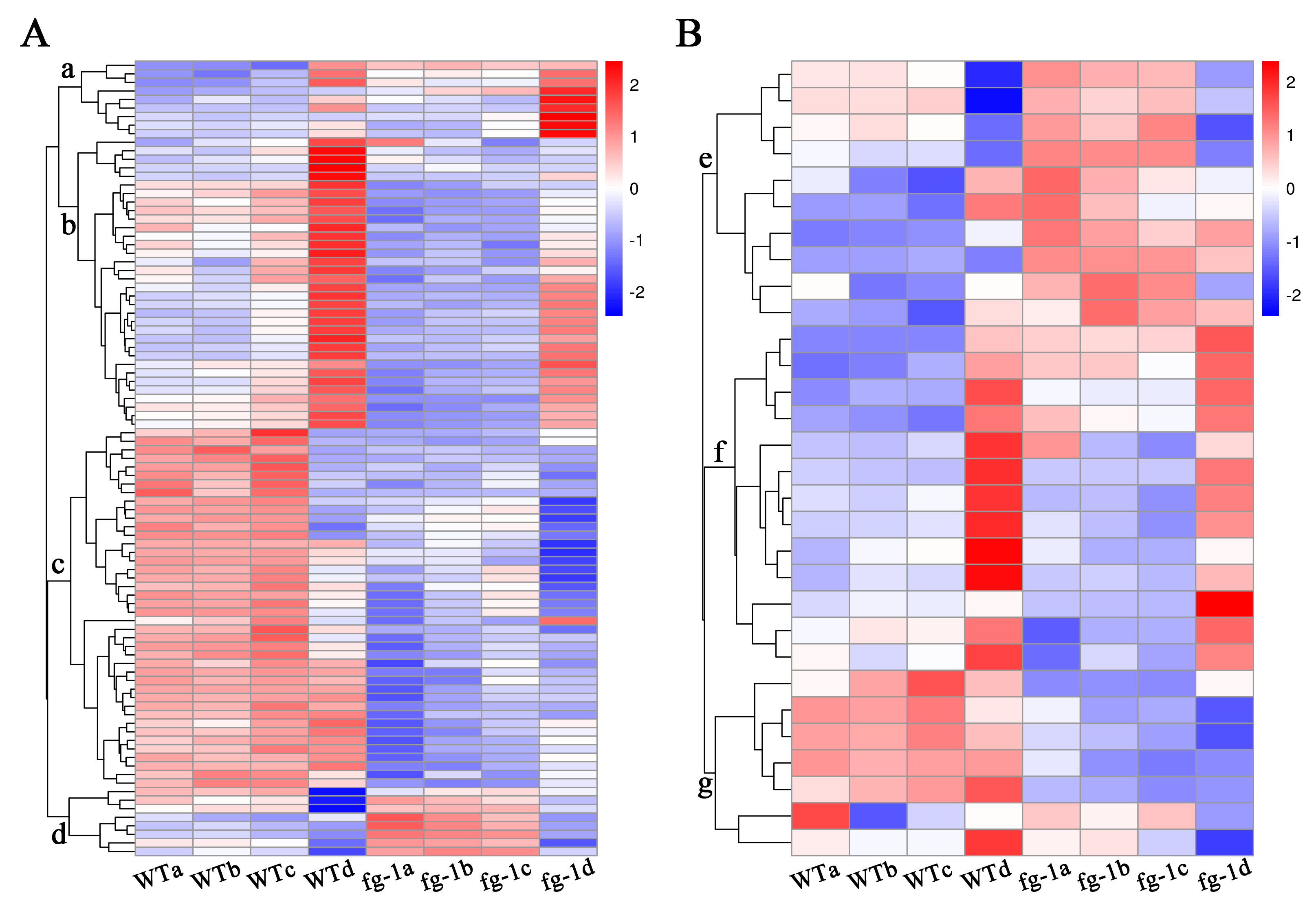

Supplement: Supplementary file 1 [file Data_Sheet_1.zip › Data Sheet 1/Supplementary Figure 6.tif]

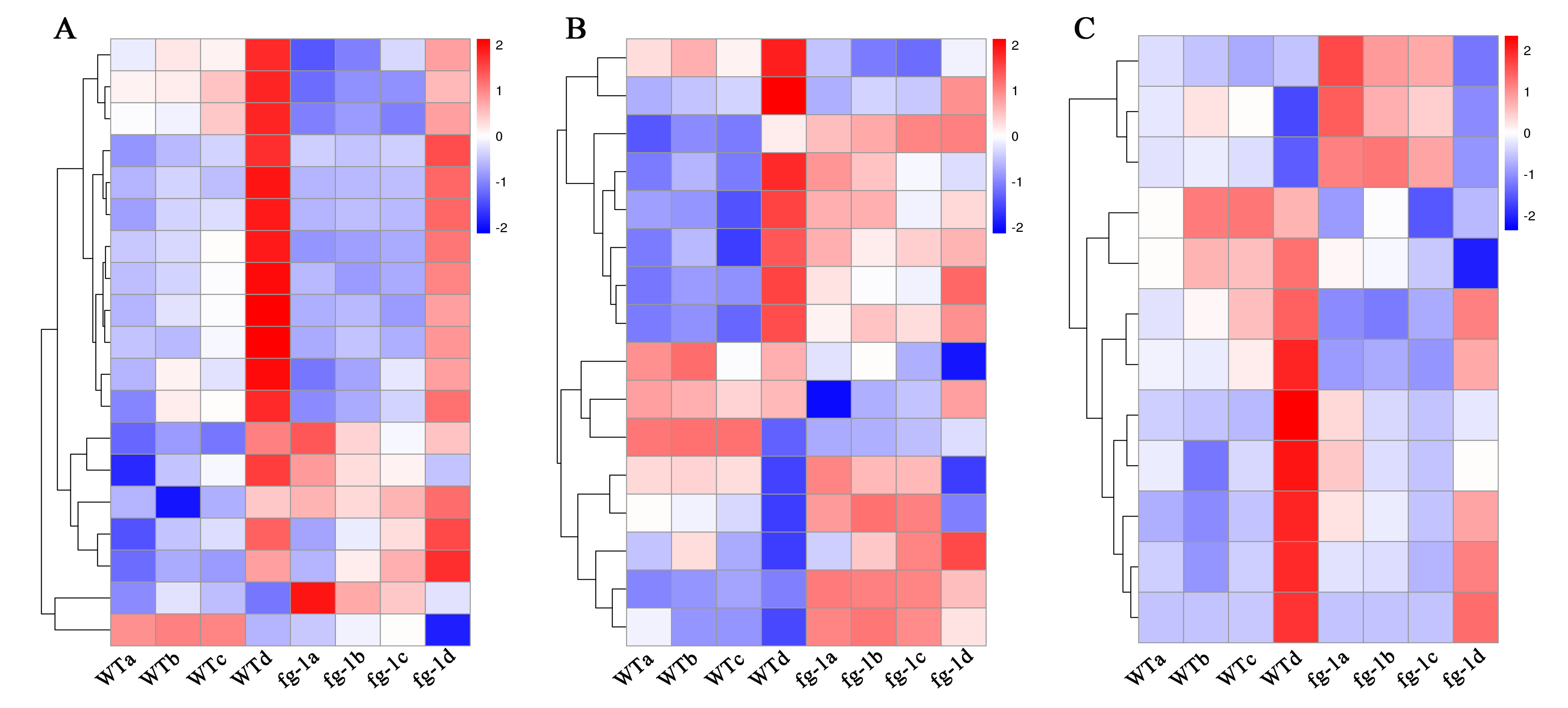

Supplement: Supplementary file 1 [file Data_Sheet_1.zip › Data Sheet 1/Supplementary Figure 7.tif]

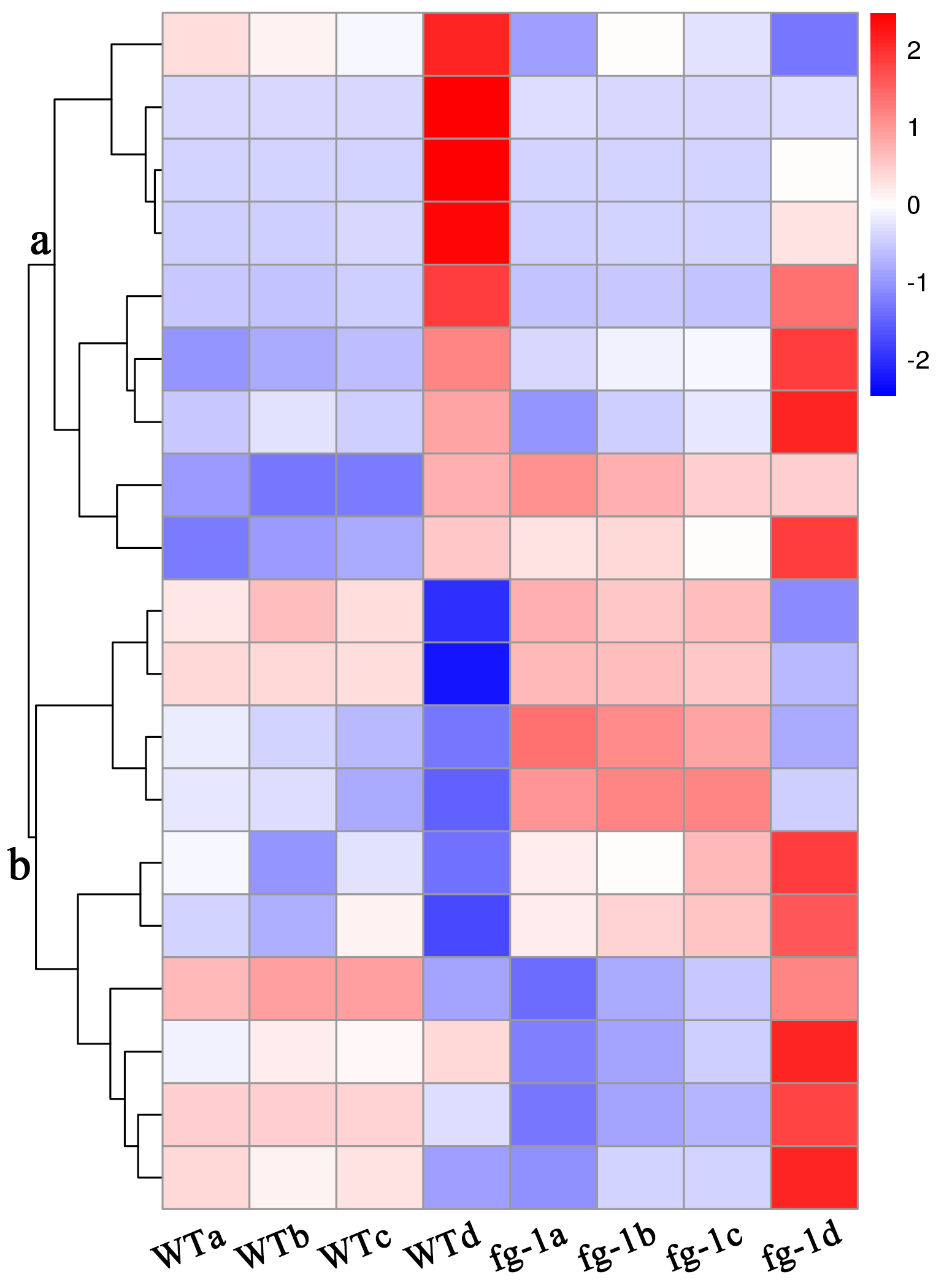

Supplement: Supplementary file 1 [file Data_Sheet_1.zip › Data Sheet 1/Supplementary Figure 8.tif]
